# Bariatric surgeries: Outcome throughout an annum at a specialist center in Malaysia

**DOI:** 10.1371/journal.pone.0285196

**Published:** 2023-05-09

**Authors:** Mustafa Mohammed Taher, Mohammed A. Abdalqader, Subhashini Jahanath, Prrasana Paramasivam, Hardip Singh Gendeh

**Affiliations:** 1 Upper Gastrointestinal and Bariatric Surgery Department, Cengild Medical Centre, Bangsar South, Kuala Lumpur, Malaysia; 2 Community Medicine Department, University of Cyberjaya, Cyberjaya, Selangor, Malaysia; 3 Hospital Kuala Lumpur, Kuala Lumpur, Malaysia; 4 Otorhinolaryngology Department, UKM Medical Center, Bandar Tun Razak, Kuala Lumpur, Malaysia; Tehran University of Medical Sciences, ISLAMIC REPUBLIC OF IRAN

## Abstract

**Introduction:**

Malaysia has the highest number of obese and overweight individuals in South-east Asia. The 2019 National Health & Morbidity survey found 50.1% of Malaysians to be overweight or obese with 30.4% being overweight and 19.7% being obese. This has led to the high rise of the bariatric surgeries demand and needs within the nation.

**Aim & objectives:**

To assess the level of fasting blood sugar (FBS), systolic, diastolic blood pressure, stop BANG score for obstructive sleep apnoea (OSA) and BMI (Body Mass Index) for the patients before and after bariatric surgeries (sleeve/gastric bypass) for a one year follow up period.

**Material & methods:**

The study was conducted among 1000 patients who underwent a single weight reduction surgery (sleeve/gastric bypass) by a single surgeon at the Cengild Medical Centre between January 2019 to January 2020. They were followed up for a one-year period through recording the parameters of fasting blood sugar (FBS), systolic, diastolic blood pressure, stop BANG score for obstructive sleep apnoea (OSA) and BMI (Body Mass Index). Study was conducted using universal sampling including all subjects visited the centre and a written consent was obtained from each participant. Descriptive statistics with mean was used and paired t-test was used for comparison and test the difference. The STOP-BANG acronym stands for: Snoring history, Tired during the day, Observed stop breathing while sleep, High blood pressure, BMI more than 35 kg/m2, Age more than 50 years, Neck circumference more than 40 cm and male Gender.

**Results:**

The mean age of patients was 38 years old. Mean FBS for the patients one month before the operation was 10.42 mmol/L and 5.84 three months post procedure. The systolic blood pressure one month before the operation and 3 months after was 139.81 mmHg and 123.79 mmHg, while diastolic blood pressure was 86.84 mmHg and 81.07 mmHg respectively. BMI was reduced from 39.69 to 27.99 after one year from the weight reduction operation. All the above parameters showed a significant reduction between one-month pre operation as compared to 3 months and 12 months post operation and that improved the health parameters of the patients significantly.

**Conclusion:**

The weight reduction operations showed a significant reduction in the FBS, blood pressure, OSA scores and BMI at 3, 12 months after the operation These patients had better overall health after the significant reduction in these parameters.

## Introduction

Malaysia has the highest number of obese and overweight individuals in South-East Asia [1]. The 2019 National Health & Morbidity survey found 50.1% of Malaysians to be overweight or obese with 30.4% being overweight and 19.7% being obese [2].

Obesity has been proven to be a risk factor for major non-communicable diseases such as Type 2 Diabetes Mellitus (T2DM), coronary heart disease, hypertension, and cancers [3]. Nyberg et al. (2018) showed a substantial decrease in the number of disease-free years in obese adults, with mild obesity (Class 1 obese) resulting in a loss of 3–4 disease-free years and severe obesity (Class 2–3 obese) causing a loss of 7–8 disease-free years [4]. The increase in obesity rates and its associated co-morbidities has caused a considerable strain to the healthcare system.

In the morbidly obese, diet and exercise alone are not enough to achieve significant weight loss. Bariatric surgery has proven to be an effective component of treatment for such groups. According to the American Society for Metabolic and Bariatric Surgery, bariatric surgery is indicated in patients with a BMI of >40, or >35 with one or more related co-morbidities such as T2DM, hypertension, and sleep apnea, among others [4].

The 2005 Asia-Pacific Bariatric Surgery Group (APBSG) consensus meeting modified the NIH indications for bariatric surgery for Asian people to recommend the procedure for those who have a BMI of >37kg/m^2^ or >32 kg/m^2^ with related co-morbidities [5]. Patients receiving bariatric surgery report not only a significant loss of excess weight after both gastric bypass and sleeve gastrectomy but also crucial improvements in obesity related NCDs; in particular, T2DM, hypertension, dyslipidemia, and obstructive sleep apnea [6].

This study with a large sample size of 1000 patients will provide a valuable addition to the bariatric literature. The demographics, co-morbidities, and post-operative outcome on weight loss and NCDs are analyzed. The study includes 1000 patients treated by a single surgeon and anesthetist, from January 2019 to January 2020 at CGI with a one-year follow-up period.

## Materials and methods

A cross sectional study was conducted among 1000 patients underwent a bariatric operation (either Sleeve/Bypass) at a specialized private referral centre for upper gastrointestinal diseases in Kuala Lumpur, Malaysia over 12 months from January 2019 to January 2020. Patient selection: for all it was Sleeve except those with significant reflux or long-standing diabetes will go Roux-en-Y Gastric Bypass (RYGB). While super obese patient will go one anastomosis gastric bypass (OAGB) [7]. Those patients followed up for a year till January 2021. Surgeries are conducted by a board-certified bariatric surgeon with more than 10 years of experience. Sampling method was universal. Informed consent was obtained which was obtained in a written form from each participant and no minors were included in that study, and ethical approval was granted from Cengild Medical Centre Ethics and Safety Committee, the approval reference number was CGI-EC-03122018-11 on the date of 03 December 2018 and data was collected during history taking at the follow up visits with the proper measurements.

A sample size of those who attended the medical center and were operating there from January 2019 to January 2020 and follow up each for a one year making a total of 1000 patients, data was obtained including the fasting blood sugar (FBS), systolic, diastolic blood pressure, stop BANG score for obstructive sleep apnoea (OSA) and BMI during the follow up visits.

The STOP-BANG acronym stands for: Snoring history, Tired during the day, Observed stop breathing while sleep, High blood pressure, BMI more than 35 kg/m2, Age more than 50 years, Neck circumference more than 40 cm and male Gender. The authors recommended that if a patient had 3 or more criteria mentioned above, it is strongly suggestive for OSA. The STOP-BANG questionnaire developed by Chung et al. has been widely used as a sensitive screening tool for OSA [8].

A descriptive statistic of mean, minimum and maximum were used in this study, in additional to inferential statistics using the paired t-test to compare between 2 groups. P value <0.05 is taken as significant level.

## Results

The 1000 patients underwent different bariatric surgeries, mainly sleeve and bypass [800 sleeves and 200 bypass (150 One anastomosis gastric bypass (OAGB), 50 Revision RYGB)]. There was a 1% drop out on the 3 months follow up, 3% drop out by 6 months follow up and total 5% by the one year follow up.

The majority of 98% of the respondents’ length of stay was of only one night and to be discharged the day after the surgical procedure, while the rest extended for 2 nights and there was less than 1% readmission.

In that study the female were the majority with 81.4% as compared to 18.6% for males. The minimum age was 28 years old as compared to maximum age of 57 years old and the average of 38.2 years old. The average fasting blood sugar (FBS) one month before the operation was 10.42 mmol/L, and one day post operation was 6.21 mmol/L, 3 months at 5.84 mmol/L and 1 year at 5.11 mmol/L.

The mean systolic blood pressure one month prior to operation was 139.81 mmHg while 3 months post operation was 123.79 mmHg and for the 12 months follow up post operation was 122.21 mmHg. Mean diastolic blood pressure one month before the operation was 86.84 mmHg while 3 months post operation was 81.07 mmHg and was 80.67 for the 12 months post operation follow up (Table 1).

**Table 1:** The descriptive statistics of the patients who underwent bariatric surgery.

	MINIMUM	MAXIMUM	MEAN	S.D.
**AGE (YEARS)**	28	57	38.2	1.8
**Glucose mmol/L (mg/dL)**				
Pre Operation	4.40 (79.28)	23.70 (427.07)	10.42 (187.76)	4.19 (75.50)
1 Day Post Operation	3.80	21.30	6.21	2.37
3 Months Post Operation	4.00	14.00	5.84	1.35
12 Months Post Operation	3.50	12.20	5.11	1.23
**Blood Pressure (mmhg)**				
Systolic Pre Operation	90.00	240.00	139.81	21.08
Diastolic Pre Operation	56.00	141.00	86.84	12.89
Systolic 3 Months Post Operation	100.00	168.00	123.79	8.86
Diastolic 3 Months Post Operation	65.00	101.00	81.07	6.12
Systolic 12 Months Post Operation	98.00	140.00	122.21	7.86
Diastolic 12 Months Post Operation	60.00	100.00	80.67	6.14
**STOP BANG SCORE**				
Pre Operation	2.00	7.00	3.95	1.09
3 Months Post Operation	0.00	4.00	1.48	0.88
12 Months Post Operation	0.00	4.00	1.36	0.85
BMI (kg/m^2^)				
Operation Day	36.42	79.90	39.69	8.65
3 Months Post Operation	32.06	63.27	33.81	7.47
6 Months Post Operation	28.98	48.23	30.62	5.66
12 Months Post Operation	23.74	51.31	27.99	6.99
**TBL (%)**				
12 Months Post Operation	28.00	39.00	35.00	2.50

The average Stop BANG score for obstructive sleep apnea (OSA) one month before the operation was 3.95 while 3 months post operation was 1.48 and for the 12 months post operation was 1.36.

BMI results was the highlight being the concern of patients. Average BMI one month pre- operation was 39.69, and 33.81 at 3 months and 27.99 at 12 months (Table 1). While the % total body loss (TBL) after 12 months follow up was on average of 35%, minimum of 28% and the maximum of 39%.

**Fig 1:**
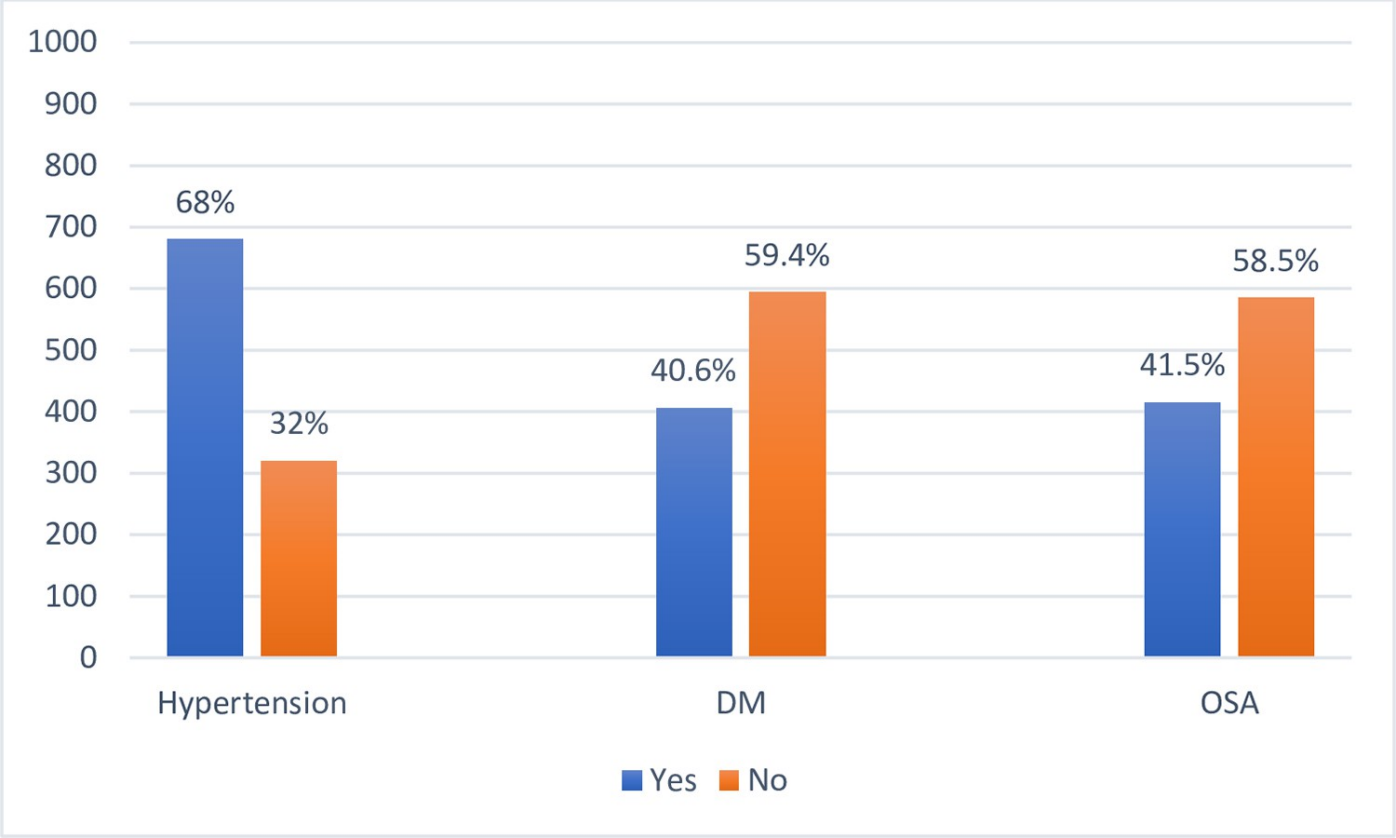
The prevalence of the comorbidities before the operation.

In Fig 1 we will see the prevalence for the comorbidities that were part of the study and was recorded before the operation as a baseline parameter, the focus was on the DM with prevalence of 40.60% and hypertension with prevalence of 68% and OSA with prevalence of 41.5%.

Those with DM, they used different treatments to control their blood sugar, and it was found that for 6.4% of them are using Insulin. While for the oral medications use, they were mainly using the Metformin (60.42%), Gliclazide (24.06%) and other medications (7.52%), while 8% were not on any diabetic medications.

While the prevalence of hypertension was 68% and they were on the following medications: 55% of them on Amlodipine, 20% on Atenolol and 15% on others antihypertensive medications and 10% of them were not on any medications.

Fig 1 also shows the prevalence of OSA among the patients underwent bariatric surgery in this study which was of 41.50%.

The bivariate association and relation between these parameters a month before the operation as compared to the same variable 3 months and 12 months after the operation using the paired t test as can be seen in Table 2. There was a significant reduction in the FBS from one month before the operation as compared to the FBS 3 months after the operation (p <0.001). The same goes for the FBS before the operation and 12 months after the operation (p <0.001), comparing the FBS among patients 3 months after the operation and 12 months after the operation also showed significant reduction. In general, it was found that one month before the operation, 40.60% of respondents were with T2DM and after one year follow up, the percentage decline to 24.70% (unchanged in the glycemic outcome after bariatric surgery), while those 159 patients (15.9%) who showed remission from diabetes, 75% of them showed complete remission which defined as normal measure of glucose metabolism Fasting Blood Sugar <100 mg/dL and 25% of them showed partial remission which is defined as subdiabetic hyperglycemia of FBS 100–125 mg/dL [9].

**Table 2:** Bivariate analysis for the patients who underwent bariatric surgery.

	MEAN DIFFERENCE	95% CONFIDENCE INTERVAL OF THE DIFFERENCE		P VALUE
		Lower	Upper	
**GLUCOSE LEVEL**				
Glucose.pre—Glucose 1day post op	1.25	0.50	2.00	0.001
Glucose pre—Glucose 3 months post op	4.99	3.85	6.13	<0.001
Glucose pre—Glucose 12 months post op	5.33	3.71	9.96	<0.001
Glucose 3 months post op—Glucose 12 months post op	0.81	0.61	1.02	<0.001
**BLOOD PRESSURE LEVEL**				
Systolic pre op–Systolic 3 months post op	17.32	14.04	20.60	<0.001
Diastolic pre op–Diastolic 3 months post op	5.76	3.73	7.79	<0.001
Systolic pre op–Systolic 12 months post op	18.91	15.68	22.14	<0.001
Diastolic pre op–Diastolic 12 months post op	6.17	4.14	8.19	<0.001
Systolic 3 months post op–Systolic 12 months post op	1.57	0.98	2.17	<0.001
Diastolic 3 months post op–Diastolic 12 months post op	0.40	0.14	0.65	0.002
**SLEEP APNEA LEVEL**				
Stop BANG pre op–stop BANG 3 months post op	2.61	2.34	2.88	<0.001
Stop BANG pre op–stop BANG 12 months post op	2.73	2.47	2.99	<0.001
Stop BANG 3 months post op–stop BANG 12 months post op	0.12	0.03	0.20	0.007
**BMI LEVEL**				
BMI pre op–BMI 3 Months post op	6.25	5.97	6.53	<0.001
BMI pre op–BMI 6 Months post op	9.13	8.53	9.73	<0.001
BMI pre op–BMI 12 Months post op	10.06	7.87	12.26	<0.001

The systolic and diastolic blood pressure showed a significant reduction between one month before the operation as compared to the readings on 3^rd^ month after the operation (p< 0.001) and the mean difference between these 2 periods was 17.32 mmHg for systolic and 5.76 mmHg for the diastolic. The reduction shown to be significant also when comparing one month before the operation with 12 months post operation and between 3 months to 12 months post operation. At the beginning of the study, it was found that 68% of the respondents were with hypertension and 60% of them showed complete remission which is defined as being normotensive (BP <120/80) off antihypertensive medication, while 20% of them went into partial remission which defined as a prehypertension values (120-140/80-89) when off medication and the other 10% of them showed improvement which was defined as a decrease in dosage or number of antihypertensive medication or decrease in systolic or diastolic blood pressure (BP) on the same medication (better control) while, the last 10% of them showed unchanged readings in the blood pressure one year after the surgical procedure [9].

The mean difference of the Stop BANG score for obstructive sleep apnea (OSA) one month before the operation compared to 3 months and 12 months post operation was statistically significant of p value < 0.001, and the mean difference between 3 months and 12 months post operation was also statistically significant of p value 0.007. OSA was noticed among 415 (41.50%) of the respondents prior to the bariatric operation and then, it was demonstrated that 218 (52.53%) of them had remission in OSA after one year following bariatric surgery.

The BMI also showed significant reduction when compare between the BMI a month before the operation which was 39.75 to 33.81 after 3 months and 27.99 after 12 months of the operation with p value <0.001 as can be seen in Table 2.

Regarding the complications trace during the one year follow up, it can be reported as follow; no major complication (no prolonged hospital stay, no reintervention, or reoperation) been witnessed. Only minor complications happened and as the following: 4 cases with marginal ulcer diagnosed with upper endoscopy, nausea and vomiting requiring intravenous fluids (IVF) among 12 patients, 1 acute renal failure managed with IVF without the need of dialysis, 3 female patients with urinary tract infection managed with antibiotics, 3 patients with dehydration requiring IV hydration as an inpatient, 1 patient with vitamin B1 deficiency and 20 patients with severe anemia requiring IV iron infusion).

## Discussion

In this study it was found the reduction in the DM, Hypertension, sleep apnea and the BMI were all significant as comparing before the bariatric surgeries and post the operation by different periods of follow ups. The basis for bariatric surgery aiming at accomplishing weight loss is the understanding that severe obesity is a disease associated with multiple health morbidities. This risk can be corrected or improved by successful weight loss in patients who were unable to achieve weight loss by non-surgical means [10].

The mechanisms by which obesity raises the risk of developing hypertension are multifactorial, involving structural, functional, and hemodynamic changes within the cardiovascular system [11]. One of the bariatric surgery outcomes is the improvement of hypertension and one of this research aims was to test the improvement in hypertension and our findings was positive to show a significant improvement in the systolic and diastolic blood pressure which was similar to another meta-analysis in which they are comparing bariatric surgery versus non-surgical treatment on blood pressure for patients with obesity covered nineteen RCTs with 1353 patients. In the pooled analyses, bariatric surgery had a significant greater effect in systolic and diastolic blood pressure reduction [12]. Some of the explanation to that reduction in blood pressure was that the restrictive and bypass bariatric surgery depresses blood pressure and plasma leptin levels within days of the procedure in both hypertensive and normotensive morbidly obese patients [13]. Therefore, bariatric surgery is an effective and viable strategy for blood pressure control in a broad population of patients with obesity and hypertension [14].

Another explanation to that reduction in the blood pressure was that the surgically induced weight loss is associated with a noticeable reduction in renal and systemic inflammation and arterial hypertension [15]. With regard to blood pressure improvement after bariatric surgery, the reasons are probably multifactorial and remain under debate, It has been speculated that a decreased inflammatory response together with an improvement in insulin resistance could reduce arterial stiffness and sodium reabsorption and hence lead to normalization of blood pressure levels [16]. Patients with central obesity are known to have increased activation of the renin–angiotensin–aldosterone system, which may also normalize after surgery [17].

In this study the remission for the hypertension after one year follow up was at level of 62% and that was close to other studies conducted on different places [18] the rest who does not get full remission could be due to the less compliance to diet plan, physical exercise and not reached the ideal weight.

Severe obstructive sleep apnea (OSA) is often seen among morbidly obese patients undergoing bariatric surgery. Snoring, STOP-Bang score ≥ 3, fatty liver, and high BMIs were significantly correlated with OSA. Dyslipidaemia and BMI were demonstrated to be associated factors for severity of OSA in this population [19]. In our study we noticed improvement in the OSA through the STOP-Bang score and that finding was consistent with another study in which they found bariatric procedures achieved profound effects on OSA, as over 75% of patients saw at least an improvement in their sleep apnoea. The remaining 25% with no improvements could be harbouring other causes of OSA such as upper airway obstruction, severe deviated nasal septum and many more. Therefore, bariatric surgery is a viable definitive treatment for obstructive sleep apnoea associated with high BMI, regardless of type [20].

This prospective multicentred study also found similar result in which they found improvements in the OSA, that study was conducted through a standard overnight cardiorespiratory recording was conducted 12 months after bariatric surgery among 132 patients who had OSA diagnosed prior to operation. The prevalence of OSA decreased from 71% at baseline to 44% at 12 months after surgery (p < 0.001). OSA was cured in 45% and cured or improved in 78% of the patients, but moderate or severe OSA still persisted in 20% of the patients after the operation [21]. Therefore, our study demonstrated that more patients had benefits from OSA improvements post procedure compared to the multicentre study above. Here we would like to suggest a referral to a sleep physician or ENT prior to procedure to ensure there are no other upper airway obstruction or confounders that may be a contributing factor to the OSA besides a high BMI.

In our study the blood glucose level was one of the independent variables of concern which of interest to show the improvement in the blood glucose level after the bariatric surgery and that’s parallel with other studies and the following also considered Type 2 diabetes mellitus (T2DM) is the most studied metabolic disorder connected with obesity, with data demonstrating that improvement and remission of T2DM in patients with obesity is superior after bariatric surgery compared with conventional medical therapy. This further solidifies that bariatric surgery is now a part of some treatment algorithm for medical management of patients with resistant T2DM and severe obesity [22]. A meta-analysis of the 11 published randomized clinical trials directly comparing bariatric/metabolic surgery versus a variety of medical/lifestyle interventions for T2DM provides evidence that surgery is superior for T2DM remission, glycemic control, and HbA_1c_ lowering. Importantly, this is equally true for patients whose baseline BMI is below or above 35 kg/m^2^ [23]. Randomized controlled clinical trials have demonstrated that bariatric surgery results in better glycemic control and greater rates of T2D remission than intensive medical/lifestyle therapy [24].

For the study limitations, as it is a single centre study, the results are not generalizable, and we will recommend doing such a study among different centres to be able to do randomization selection to generalize the results.

## Conclusion

This study shows that bariatric surgery at a specialized referral center with dedicated upper gastrointestinal services does have improved outcomes as the decline in the FBS, blood pressure, OSA and BMI were all significant and that will support the conclusion of the effectiveness of the bariatric surgeries in managing the metabolic morbidities that been studied.
